# Fact-Checking Cancer Information on Social Media in Japan: Retrospective Study Using Twitter

**DOI:** 10.2196/49452

**Published:** 2023-09-06

**Authors:** Nari Kureyama, Mitsuo Terada, Maho Kusudo, Kazuki Nozawa, Yumi Wanifuchi-Endo, Takashi Fujita, Tomoko Asano, Akiko Kato, Makiko Mori, Nanae Horisawa, Tatsuya Toyama

**Affiliations:** 1 Department of Breast Surgery Nagoya City University Graduate School of Medical Sciences Nagoya Japan; 2 Department of Breast Oncology Aichi Cancer Center Hospital Nagoya Japan

**Keywords:** cancer, fact-check, misinformation, social media, twitter

## Abstract

**Background:**

The widespread use of social media has made it easier for patients to access cancer information. However, a large amount of misinformation and harmful information that could negatively impact patients’ decision-making is also disseminated on social media platforms.

**Objective:**

We aimed to determine the actual amount of misinformation and harmful information as well as trends in the dissemination of cancer-related information on Twitter, a representative social media platform. Our findings can support decision-making among Japanese patients with cancer.

**Methods:**

Using the Twitter app programming interface, we extracted tweets containing the term “cancer” in Japanese that were posted between August and September of 2022. The eligibility criteria were the cancer-related tweets with the following information: (1) reference to the occurrence or prognosis of cancer, (2) recommendation or nonrecommendation of actions, (3) reference to the course of cancer treatment or adverse events, (4) results of cancer research, and (5) other cancer-related knowledge and information. Finally, we selected the top 100 tweets with the highest number of “likes.” For each tweet, 2 independent reviewers evaluated whether the information was factual or misinformation, and whether it was harmful or safe with the reasons for the decisions on the misinformation and harmful tweets. Additionally, we examined the frequency of information dissemination using the number of retweets for the top 100 tweets and investigated trends in the dissemination of information.

**Results:**

The extracted tweets totaled 69,875. Of the top 100 cancer-related tweets with the most “likes” that met the eligibility criteria, 44 (44%) contained misinformation, 31 (31%) contained harmful information, and 30 (30%) contained both misinformation and harmful information. Misinformation was described as Unproven (29/94, 40.4%), Disproven (19/94, 20.2%), Inappropriate application (4/94, 4.3%), Strength of evidence mischaracterized (14/94, 14.9%), Misleading (18/94, 18%), and Other misinformation (1/94, 1.1%). Harmful action was described as Harmful action (9/59, 15.2%), Harmful inaction (43/59, 72.9%), Harmful interactions (3/59, 5.1%), Economic harm (3/59, 5.1%), and Other harmful information (1/59, 1.7%). Harmful information was liked more often than safe information (median 95, IQR 43-1919 vs 75.0 IQR 43-10,747; *P*=.04). The median number of retweets for the leading 100 tweets was 13.5 (IQR 0-2197). Misinformation was retweeted significantly more often than factual information (median 29.0, IQR 0-502 vs 7.5, IQR 0-2197; *P*=.01); harmful information was also retweeted significantly more often than safe information (median 35.0, IQR 0-502 vs 8.0, IQR 0-2197; *P*=.002).

**Conclusions:**

It is evident that there is a prevalence of misinformation and harmful information related to cancer on Twitter in Japan and it is crucial to increase health literacy and awareness regarding this issue. Furthermore, we believe that it is important for government agencies and health care professionals to continue providing accurate medical information to support patients and their families in making informed decisions.

## Introduction

There are 4.76 billion social media users around the world, equating to approximately 60% of the total global population [1]. The number of individuals that rely on social media as a source of information has been increasing. In a worldwide survey conducted in 2020, more than 65% of respondents from populous countries declared that they rely on social media as a source of news [2]. Twitter is one of the most popular social media platforms globally, with more than 330 million monthly active users worldwide [3] and 59 million in Japan in 2023 [4].

The Twitter platform allows anyone to post messages, or “tweets,” freely and conveniently, and a great deal of cancer-related information is disseminated on this social media service. The spread of social media has made it easier to acquire information about cancer; thus, many patients use social media platforms for this purpose, including Twitter. Numerous studies have reported various potential advantages for patients with cancer from using social media [5]. However, potential disadvantages associated with social media usage by patients have also been reported in the literature [5]. The reliability and quality of web-based health information cannot always be trusted, and this information should not be used as a substitute for professional medical advice. It is essential to note that many web-based sources present medically incorrect, ambiguous, and risky treatment options and guidance [6-8]. Unfortunately, not only factual information but also a large amount of misinformation is spread via social media [9]. In Japan, fewer hospitals and clinics use social media in comparison with other countries because some content disseminated by these medical institutions’ conflicts with information in medical advertising [10]. This state of affairs can lead to an increased prevalence of inaccurate information on social media platforms. Additionally, cultural differences can affect choices regarding actions in seeking information related to cancer treatment. Japanese patients tend to rely on media and commercial resources more than White and non–Japanese Asian patients [11]. However, the types of cancer-related information being disseminated on social media in Japan are unclear.

The objective of our research was to examine the prevalence of misinformation and harmful cancer-related content on Twitter and to further clarify attributes that increase the likelihood of the dissemination of such content in Japan. The findings of our study can assist decision-making among individuals diagnosed with cancer.

## Methods

### Data Acquisition and Selection of Tweets Containing Cancer Information

Tweet data were retrospectively collected from August 2022 to September 2022 by querying the Twitter app programming interface with the keyword “cancer” in Japanese using Jupyter Notebook (version 6.3.0; Project Jupyter) [12] referred to example codes supplied by Twitter Developer Platform [13]. Query was conducted on September 29, 2022. The Twitter data set contained tweet-level data including the date or time, account’s screen name, tweet description, number of the account’s followers, “likes” count, and retweet count at the time of data acquisition. The eligibility criteria were original tweets or retweets with comments with the following information: (1) reference to a causal relationship regarding the occurrence or prognosis of cancer; (2) recommendation or nonrecommendation of actions for the general public, patients with cancer, or health care professionals; (3) reference to the course of cancer treatment or adverse events; (4) results of cancer research; and (5) other cancer-related knowledge and information. The exclusion criteria were as follows: retweets without any comments, and tweets with content unrelated to cancer topics (Multimedia Appendix 1). On the review process, we first narrowed down all the acquired tweets to the 2000 tweets with the most “likes” to facilitate the review process because our final goal is to extract the 100 tweets with the most “likes.” We reviewed all 2000 tweets and the eligible tweets were selected based on eligibility criteria and exclusion criteria. The 100 tweets with the most “likes” were finally extracted from the eligible tweets.

### Accuracy and Harm Analysis

We followed the method reported by Johnson et al [9] in the analysis of accuracy and harm with respect to cancer-related web-based information. Two independent reviewers, who were physicians specializing in oncology and with a clinical practice in a cancer center or university hospital in Japan, reviewed the medical claims in each tweet and completed 4-question assessments adapted from assessments of factuality and credibility through an iterative process with NK, MK, MT, and KN. In the analysis of accuracy, the reviewers scored each tweet from 1 to 5 points (1: true, 2: mostly true, 3: both true and false, 4: mostly false, and 5: false). The average of the 2 physicians was rounded to the nearest whole number, with scores of 3 or more indicating misinformation and scores of 2 or less indicating factual information. The reviewers provided the reasons for selecting a score of 3 or higher as follows: Unproven, Disproven, Inappropriate application, Strength of evidence mischaracterized, Misleading, and Other misinformation; multiple selections were allowed. In harm analysis, the reviewers gave each tweet a score from 1 to 5 (1: Definitely not harmful, 2: Probably not harmful, 3: Uncertain, 4: Probably harmful, and 5: Definitely harmful). Harmful information was defined as any rating by at least one reviewer of “Probably harmful” or “Definitely harmful”; the remaining options were classified as Safe information. The reviewers provided the reasons for selecting “Probably harmful” and “Definitely harmful” for the content, as follows: Harmful action, Harmful inaction, Harmful interactions, Economic harm, and Other harmful information, with multiple selections allowed. The level of agreement between raters was assessed using the Cohen κ coefficient. We calculated the proportion of tweets identified as containing misinformation and harmful information and analyzed reviewers’ explanations for the ratings, including for multiple selections.

### Ethics Approval

The first protocol for this study (protocol no. 60-22-0148) was approved by the institutional review board of Nagoya City University Graduate School of Medical Sciences in April 2023.

### Statistical Analysis

To ascertain the extent to which cancer-related information was disseminated, we evaluated the number of times a tweet was reposted on Twitter (ie, retweeted). The Mann-Whitney *U* test was used to compare the number of “likes” and retweets for the leading 100 tweets. All figure creation and statistical analyses were performed using Prism (version 9.0.0; GraphPad LLC). *P*<.05 was considered to indicate a significant difference.

## Results

**Figure 1:**
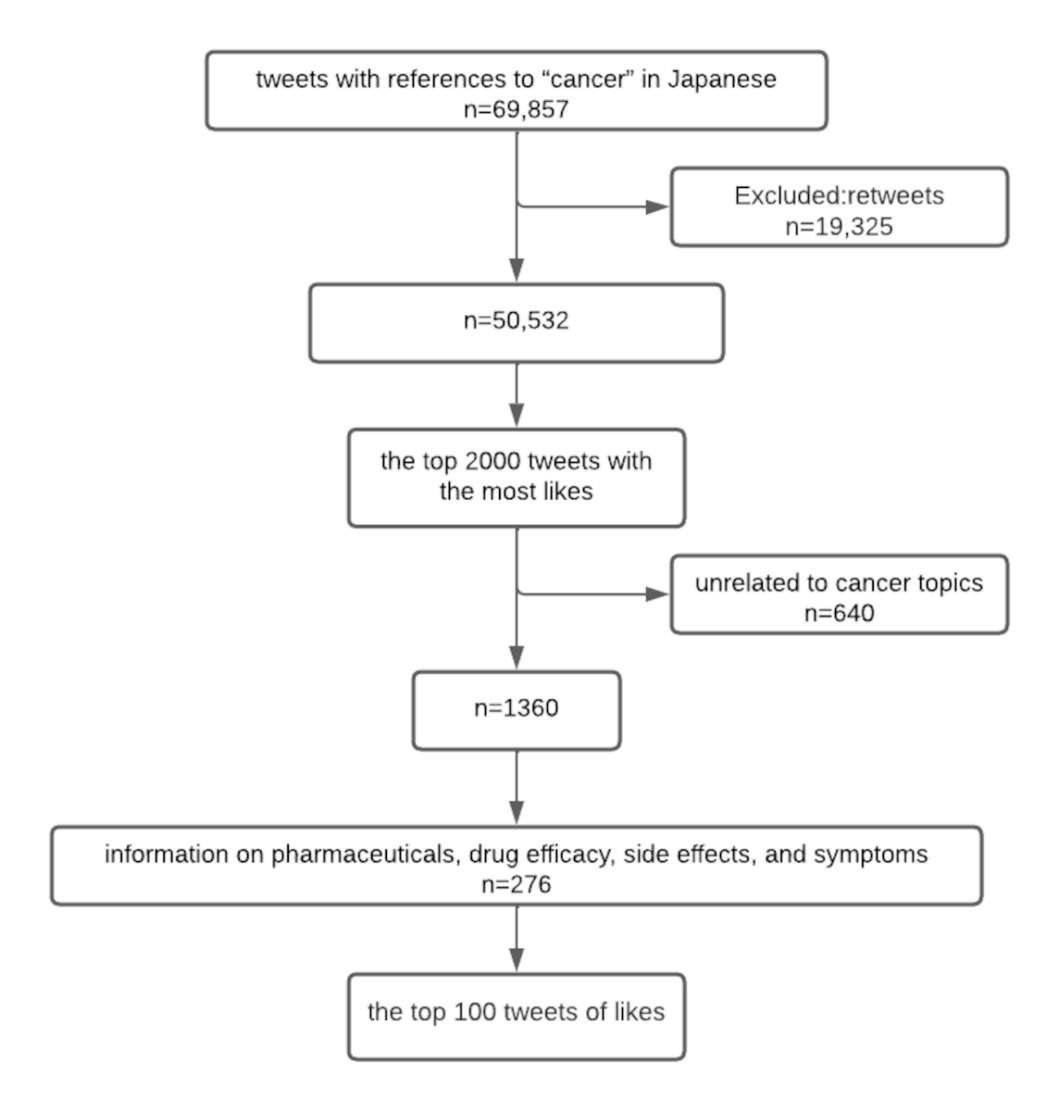
CONSORT (Consolidated Standards of Reporting Trials) diagram of this study.

The CONSORT (Consolidated Standards of Reporting Trials) diagram for this study is shown in Figure 1. A total of 69,857 tweets in Japanese with references to “cancer” were identified. Of those, 19,325 tweets were excluded because they were retweets. We narrowed down the 50,532 remaining tweets to the 2000 tweets with the most “likes.” We excluded tweets with content unrelated to cancer topics, which resulted in 1360 tweets. For these, the selection criteria were set to include information on pharmaceuticals, drug efficacy, side effects, and symptoms, resulting in 276 tweets selected. We chose the 100 tweets with the most “likes” from among these 276 tweets (details of the top 100 tweets are shown in Multimedia Appendix 2).

**Figure 2:**
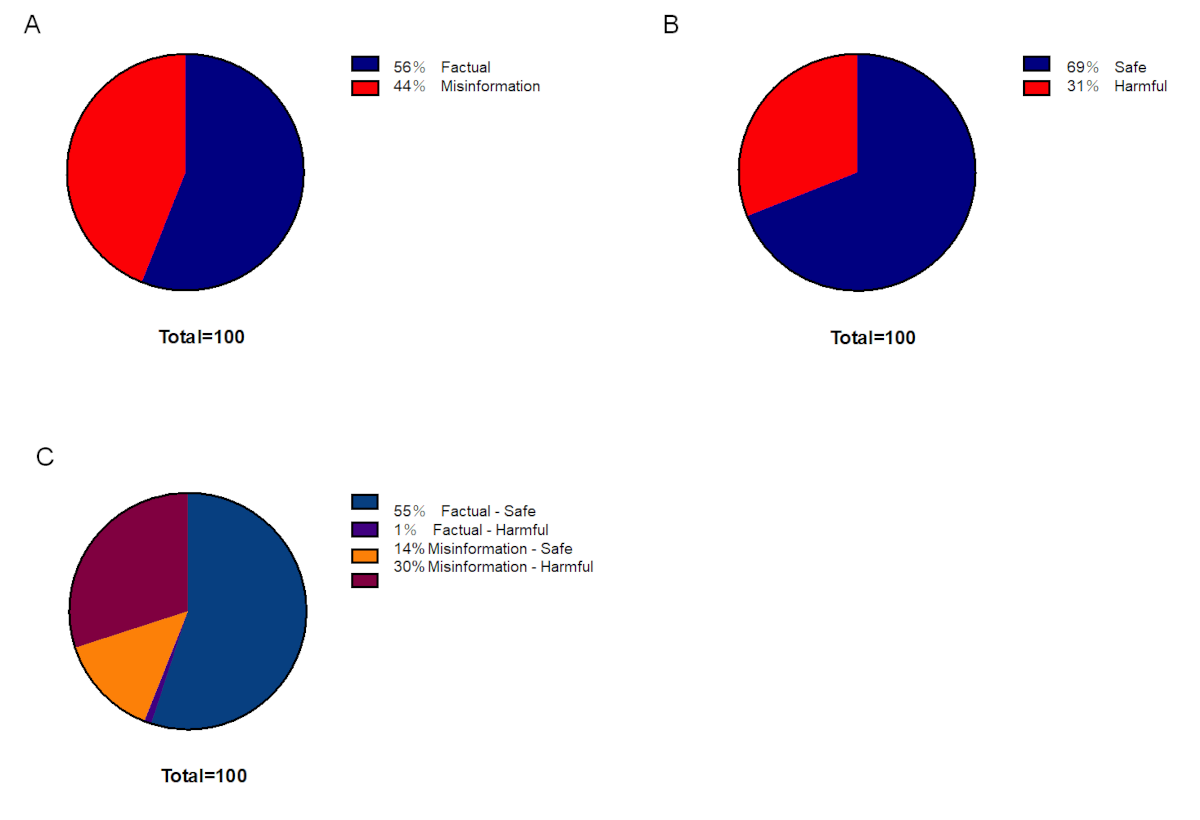
Proportion of factual or misinformation, and safe or harmful information in the leading 100 tweets. (A) Factual or Misinformation, (B) Safe or Harmful, and (C) 2×2 combination.

Following expert review, 44% of the leading 100 tweets contained misinformation (n=44; κ=0.50, 95% CI 0.38-0.65; Figure 2A), described as Unproven (29/94, 40.4%), Disproven (19/94, 20.2%), Inappropriate application (4/94, 4.3%), Strength of evidence mischaracterized (14/94, 14.9%), Misleading (18/94, 18%), and Other misinformation (1/94, 1.1%; Table 1). In total, 31% of tweets were classified as containing harmful information (n=31; κ=0.62, 95% CI 0.44-0.79; Figure 2B), described as Harmful action (9/59, 15.2%), Harmful inaction (43/59, 72.9%), Harmful interactions (3/59, 5.1%), Economic harm (3/59, 5.1%), and Other harmful information (1/59, 1.7%; Table 2). Each example of misinformation and harmful information are shown in Multimedia Appendix 3.

**Table 1:** Reasons for reviewers’ rating tweets as misinformation.

Misinformation	Tweets, n (%)
Unproven	38 (40.4)
Disproven	19 (20.2)
Inappropriate application	4 (4.3)
Strength of evidence mischaracterized	14 (14.9)
Misleading	18 (19.1)
Other	1 (1.1)
Total	94 (100)

**Table 2:** Reasons for reviewers’ rating tweets as harmful information.

Harmful information	Tweets, n (%)
Harmful action	9 (15.2)
Harmful inaction	43 (72.9)
Harmful interactions	3 (5.1)
Economic harm	3 (5.1)
Other	1 (1.7)
Total	59 (100)

**Figure 3:**
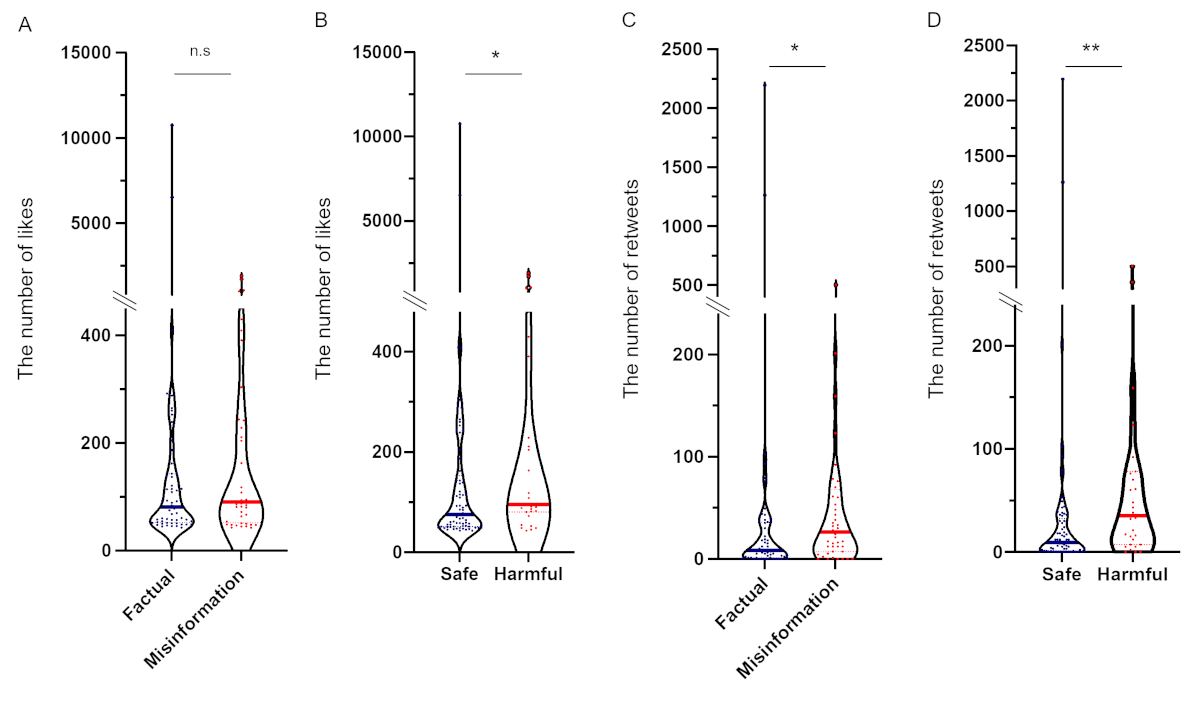
Comparison of the total number of likes and retweets for the leading 100 tweets. Number of likes for (A) Factual or Misinformation, and (B) Safe or Harmful information. Number of retweets for (C) Factual or Misinformation, and (D) Safe or Harmful information. The Mann-Whitney *U* test was used to compare the number of likes and retweets of the top 100 tweets (n.s: not significant; *P<.05; **P<.01). Median is shown as solid lines and quartiles as dashed lines.

In the analysis of accuracy and harm combined, the proportions of tweets rated as Factual-Safe, Factual-Harmful, Misinformation-Safe, and Misinformation-Harmful were 55% (55/100), 1% (1/100), 14% (14/100), and 30% (30/100), respectively (Figure 2C). A high concordance rate between Misinformation and Harmful information was observed, and 68.9% (30/44) of tweets containing misinformation included harmful information. The median number of “likes” for the top 100 tweets was 76.5 (IQR 43-10,747). The number of “likes” for Factual information (median 93, IQR 45-10,747) and for Misinformation (median 92.5, IQR 43-1919) were statistically comparable (*P=*.24; Figure 3A). Harmful information (median 95, IQR 43-1919) was liked more often than safe information (median 75.0, IQR 43-10,747; *P*=.04; Figure 3B). The median number of retweets for the leading 100 tweets was 13.5 (IQR 0-2197). Misinformation was disseminated significantly more often than Factual information (median 29.0, IQR 0-502 vs 7.5, IQR 0-2197; *P*=.01; Figure 3C). Harmful information was also disseminated significantly more often than Safe information (median 35.0, IQR 0-502 vs 8.0, IQR 0-2197; *P*=.002; Figure 3D).

## Discussion

### Principal Results

We retrospectively collected data from the Twitter social media platform in Japan and conducted fact-checking with independent experts. Our study revealed that many tweets containing misinformation and harmful information were widely disseminated in Japanese on Twitter. This was the first study to demonstrate that misinformation and harmful information is easily spread on Twitter in Japan.

In our study, we found that 44% (44/100) of the top 100 tweets in Japanese containing cancer-related information included misinformation. Among the misinformation in this study, the content that received the highest number of “likes” and retweets was related to the notion that “in Japan, cancer mortality rates are high due to the importation of surplus and discarded anticancer drugs from the United States.” Despite the absence of such facts, it has garnered significant engagement in terms of “likes” and retweets. The prevalence of misinformation on the web has become a serious issue, and it is of particular concern in the area of medical information. A systematic review of 69 studies on medical misinformation on the Internet found that Twitter had a high rate of misinformation, especially with regard to drugs such as tobacco and opioids [14]. In that systematic review, some studies reported that up to 87% of the information was inaccurate. In particular, approximately 40% of the information related to cancer was found to be misinformation, which is equivalent to the rates of misinformation on vaccines and eating disorders. However, most past studies have focused on English-language articles, and there is insufficient research on differences across languages regarding the accuracy of information on social media. In Japan, it has been reported that among Japanese web search results for cancer screening, 52% of the top-ranked websites supported anticancer screening claims [15]. These websites, which often hinder cancer screening and treatment based on unscientific claims by antiscreening or anticancer treatment influencers, present a serious problem as they garner strong support among those who are skeptical of science. However, few reports have mentioned medical information on social media in Japan, especially cancer information. Owing to the possibility that content distributed by hospitals and clinics may violate medical advertising regulations, past reports have suggested that social media is relatively less used in Japan than in other countries [10] and that the environment surrounding medical information on the Internet in Japanese language is unfavorable. Our study suggested that the level of cancer misinformation in Japan is comparable to that in countries where mainly English is spoken [9]. The present results confirm the unfavorable environment related to cancer information on social media in Japan.

We also evaluated the safety of the information disseminated on Twitter. We found that 31% of tweets contained harmful content, and 68.9% of tweets containing misinformation included harmful information. Moreover, harmful information was preferred by Twitter users and was shared more readily than safe information. The most frequently cited reason that medical experts considered the information to be harmful was owing to harmful inaction (72.9% of tweets), followed by harmful action. Harmful inaction discourages people from engaging in certain recommended actions related to health care. Examples of harmful inaction in our study included messages that urged individuals to abstain from receiving the human papillomavirus (HPV) vaccine or advised against undergoing chemotherapy. The topic of HPV vaccines has been reported in relation to widespread misinformation on the web [16-18]. In Japan, following unconfirmed reports of unusual postvaccination symptoms, the Japanese Ministry of Health, Labour, and Welfare suspended its proactive recommendations for vaccine and stop promoting the use of the vaccines from 2013 to 2022 [19]. However, it has been demonstrated that the HPV vaccination was not significantly associated with the incidence of unusual postvaccination symptoms [20]. The cessation of recommendations for vaccine led to a dramatic decrease in vaccination coverage and that was estimated to result in excess deaths from cervical cancer in Japan [21]. Thus, misinformation on social media poses an issue that cannot be disregarded. Additionally, misinformation may include content that advocates alternative therapies instead of standard treatment for curable cancers. For instance, there were tweets asserting that cancer can be improved solely through arm-swinging exercise or claiming that cannabis has anticancer effects. In fact, it has been reported that patients who receive alternative therapies for which there is insufficient evidence have a poorer prognosis than patients who receive standard treatment [22,23], highlighting the potential impact of access to accurate information on cancer outcomes. Our findings are consistent with previous research demonstrating that misinformation has a greater propensity to be propagated than factual information. Within the medical news domain, unsubstantiated information has been shown to garner more attention than accurate information [24], and false and harmful information spreads more easily [9]. The reasons for the dissemination of misinformation are not always clear and may be attributable to habitual web-based information-sharing. Intriguingly, one study indicated that individuals who frequently shared false information were also likely to share true information, and those who shared politically left-leaning news also tended to share right-leaning news [24]. This suggests that motivation alone cannot fully explain the propagation of misinformation, which may be indicative of a lack of critical thinking beyond mere bias. It is also reported that the incentivization of “likes” and comments could potentially alter the information-sharing behavior of the disseminator.

Another potential reason for the high level of misinformation is the lack of posts by public health organizations and health care providers. As described above, medical professionals in Japan do not use social media to disseminate health care information [10]. Although misinformation tends to propagate easily, it is unlikely that the current situation will improve unless accurate information is also shared on social media platforms. Because nonmedical professionals who seek cancer information frequently use social media, it has become necessary for health care professionals and institutions to officially establish a presence on social media to disseminate accurate medical information in Japan.

### Limitations

The main limitation of this study is that this was a retrospective trial, which may result in selection bias. However, efforts were made to minimize information bias by excluding tweets related to the Breast Cancer Awareness Month campaign and setting the data collection period to exclude Pink Ribbon Week. We also discovered that a cancer eradication campaign was conducted from August to September 2022, and tweets mentioning this campaign were excluded from the analysis. Additionally, the short data collection period (2 months) and limited number of included tweets are also considered limitations. Despite high variability in the number of “likes” and retweets among the leading 100 tweets, we obtained meaningful results by excluding outliers. Another limitation is the subjective judgment regarding the accuracy and harmfulness or safety of the information, which was addressed by 2 reviewers evaluating each tweet independently to ensure objectivity.

### Conclusions

We demonstrated a high prevalence of misinformation and harmful information related to cancer on Twitter in Japan. It is crucial to improve health literacy by raising awareness about the prevalence of cancer-related misinformation. Continued dissemination of accurate medical information by governments and health care professionals can support decision-making for patients and their families. Further research is required to identify those individuals who are actively involved in the dissemination of cancer misinformation; assess the extent of its influence on scientific beliefs, trust, and decision-making processes; and explore the potential impact of physician-patient interactions in rectifying any misinformation.

## Appendix

Multimedia Appendix 1 Inclusion or exclusion criteria.

Multimedia Appendix 2 Detail of the top 100 tweets.

Multimedia Appendix 3 Examples of misinformation and harmful information.

## Abbreviations

CONSORT: Consolidated Standards of Reporting Trials
HPV: human papilloma virus

## Data Availability

The data sets generated and analyzed during this study are available.
