# Supplementary material for: Fact-Checking Cancer Information on Social Media in Japan: Retrospective Study Using Twitter
Source: JMIR Form Res. 2023 Sep 6;7:e49452. doi: 10.2196/49452 (PMC10512120; doi:10.2196/49452)
Supplement: Multimedia Appendix 1 [file formative_v7i1e49452_app1.docx]

**Multimedia Appendix 1.** Inclusion or Exclusion criteria.

| **Inclusion criteria**   - Tweets with comment containing the term “cancer” in Japanese - Original tweets or retweets with comments with the following information:   1) reference to a causal relationship regarding the occurrence or prognosis of cancer  2) recommendation/non-recommendation of actions for the general public, patients with cancer, or health care professionals  3) reference to the course of cancer treatment or adverse events  4) results of cancer research  5) other cancer-related knowledge and information  **Exclusion criteria**   - Retweets without any comments - Tweets with content unrelated to cancer topics |
| --- |
