# Supplementary material for: Fact-Checking Cancer Information on Social Media in Japan: Retrospective Study Using Twitter
Source: JMIR Form Res. 2023 Sep 6;7:e49452. doi: 10.2196/49452 (PMC10512120; doi:10.2196/49452)
Supplement: Multimedia Appendix 3 [file formative_v7i1e49452_app3.docx]

**Multimedia Appendix 2.** Examples of misinformation and harmful information.

| Information type | Category | Example |
| --- | --- | --- |
| Misinformation | Unproven (eg, not studies, insufficient evidence) | Arm swing exercises can improve cancer. |
|  | Disproven (eg, studies and ineffective) | An increase in the HPV vaccination rates has been accompanied by a rise in the incidence of cervical cancer.^a^ |
|  | Inappropriate application (eg, wrong disease site, misapplication of preclinical data) | Tofu, being rich in high-quality proteins and abundant in B-vitamins is effective in cancer prevention |
|  | Strength of evidence mischaracterized (eg, weak evidence portrayed as strong or vice versa) | Cruciferous vegetable can prevent 20%-30% of cancer. |
|  | Misleading (eg, title not supported by text, statics/data do not support conclusion) | The contents of HPV vaccine, COVID-19 vaccine, and influenza vaccine are all the same. |
| Harmful information | Harmful action (eg, potentially toxic effects of doing the suggested test or treatment) | Cannabinoids have anticancer properties. |
|  | Harmful inaction (eg, could lead to a delay or not seeking medical attention for treatable/curable condition) | Chemotherapy is harmful. |
|  | Harmful interactions (eg, known/unknown medical interactions with curative therapies) | Carrot juice, wild grass teas, and fermented food are recommended for patients with cancer. |
|  | Economic Harm (eg, out of financial costs associated with treatment/travel) | Cruciferous vegetable can prevent 20%-30% of cancer. |

^a^HPV: human papilloma virus.
